# Intracellular chemical gradients: morphing principle in bacteria

**DOI:** 10.1186/2046-1682-5-18

**Published:** 2012-09-07

**Authors:** Robert G Endres

**Affiliations:** 1 Division of Molecular Biosciences & Centre for Systems Biology and Bioinformatics, Imperial College, London, SW7 2AZ, UK

## Abstract

Advances in computational biology allow systematic investigations to ascertain whether internal chemical gradients can be maintained in bacteria – an open question at the resolution limit of fluorescence microscopy. While it was previously believed that the small bacterial cell size and fast diffusion in the cytoplasm effectively remove any such gradient, a new computational study published in *BMC Biophysics* supports the emerging view that gradients can exist. The study arose from the recent observation that phosphorylated CtrA forms a gradient prior to cell division in *Caulobacter crescentus*, a bacterium known for its complicated cell cycle. Tropini et al. (2012) postulate that such gradients can provide an internal chemical compass, directing protein localization, cell division and cell development. More specifically, they describe biochemical and physical constraints on the formation of such gradients and explore a number of existing bacterial cell morphologies. These chemical gradients may limit in vitro analyses, and may ensure timing control and robustness to fluctuations during critical stages in cell development.

## Commentary

**Figure 1:**
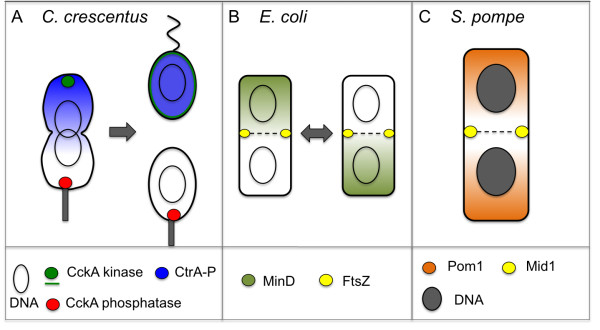
**Examples of intracellular chemical gradients in microbes.** ( **A**) CtrA-P gradient in *C. crescentus* determines asymmetric cell division into swarmer cell (top daughter) and stalked cell (bottom daughter). ( **B**) Alternating MinD gradients (oscillations) determine FtsZ recruitment at midcell and hence the cell-division plane in *E. coli*. ( **C**) Pom1 gradient in fission yeast has a similar function to Min system in bacteria. Relative cell sizes ( **A-C**) are not drawn to scale.

Bacteria keep surprising us: they are social and communicate with each other [1], and they are well-organised internally using cytoskeleton-protein homologues from larger eukaryotic cells [2]. Now the current study explores the conditions under which bacterial cells can establish spatial chemical gradients, potentially acting similarly to morphogenic fields for differentiation in animal embryos [3,4]. Examples are potentially widespread: phosphorylated CtrA (CtrA-P) in *C. crescentus*[5], virulence protein IcsA in *Shigella flexneri*[6], as well as MinCD proteins for determining midcell for cell division in *Escherichia coli* and *Bacillus subtilis*[7]. MinC functions similarly to kinase Pom1 in the significantly larger *Schizosaccharomyces pombe* (fission yeast) by inhibiting cell division and hence controlling cell size [8-10] (see Figure 1). Intracellular gradients seem to matter and sometimes also need to be suppressed. For instance, in *E. coli* chemotaxis [11,12] the internal gradient of CheY-P is suppressed by co-localising the phosphatase with the kinase at the receptor cluster so all flagella encounter the same concentration of the response regulator [13]. Hence, internal chemical gradients are not so unusual after all. Already 60 years ago, Alan Turing proposed concentration patterns in non-equilibrium steady states [14,15] – states which often surprise our equilibrium-physics trained minds.

Previously, it was believed that chemical gradients inside a small micron-sized bacterial cell are quickly wiped out by fast diffusion. This is similar to the notion that bacteria are unable to directly sense external spatial gradients, only temporally by comparison measurements while swimming. However, such subtle questions are difficult to assess, and this is where computational biology can help [16]: computational analysis can guide difficult experiments and allow us to scan through multiple parameters in search for interesting mathematical solutions. For instance, such analysis showed that spatial chemical gradients can, in principle, be sensed by bacteria after all [17,18] and an example was later found [19]. The exploration of the physical limits of sensing was then also extended to sensing of external chemical gradients with unexpected predictions [20].

Tropini et al. [21] ask under what conditions a localised source and sink on two cell ends can lead to a significant chemical gradient. Extending previous studies by others [22], they systematically explored different cell geometries, including round, rod-shaped, curved, Y-shaped and dividing cells. They also investigated biochemical effects such as enzyme localization and saturation, and explored large parameter regimes to address robustness. Their principal finding is that gradients can exist as long as the kinetics of the source and sink are on timescales faster than the typical time required to diffuse across the length of the cell. However, due to restrictions from numerical solutions of the reaction-diffusion equations, no linear stability analysis could be done, e.g. to see how gradients respond to perturbations. Additionally, due to the continuum approximation for molecular concentrations, no stochastic fluctuations could be included to see how molecular noise affects gradients [23]. Does this lessen the impact of the paper? – In light of the advanced computational algorithms used, we would argue no. The authors offer a systematic exploration of parameter values to investigate robustness (and effectively the influence of noise on a gradient), and connect to actual biological examples from various bacterial species. Furthermore, while other molecular components and structures such as chromosomes were neglected, molecular crowding would actually favour the establishment of gradients by increasing the time scale for movement between the poles.

If cell division provides asymmetry anyway, e.g. via old and new cell poles, why deploy gradients prior to cell division? For instance, in *C. crescentus* second messanger cyclic-di-GMP is asymmetrically distributed between the two daughter cells right after cell division for motility and organelle formation [24]. The authors propose an intriguing possibility. Intracellular gradients may ensure timing control and robustness to fluctuations during the life of the cell prior to cell division. Bacteria and fission yeast, both of which have cell walls, need to actively regulate cell division - otherwise widespread cell geometries and large fluctuations would be unavoidable [25]. Utilising a gradient might also be better for cell-size control than a threshold-crossing mechanism of a uniformly distributed protein using dilution during growth [26]. Of practical importance for the experimentalist, these chemical gradients may limit the usefulness of in vitro analyses since concentrations are homogenised. However, the study also cautions in vivo analyses using GFP-tagged proteins, as changing the size of proteins may alter their diffusion constants [27].

## Conclusions

Lewis Wolpert had the foresight that morphogen gradients can provide the necessary positional information for structuring the developing embryo [28,29]. The current study extends this powerful idea to tiny bacteria. Furthermore, the results by Tropini et al. highlight the utility of mathematical modelling in future studies of intracellular organization in bacteria, and illustrate the complex spatial patterning that can be achieved even in the absence of membrane compartmentalization.

## Competing interests

The authors declare no competing interests.

## Authors’ contributions

RGE conceived and wrote manuscript.
